# Efficacy of treatment for acneiform eruptions related to epidermal growth factor receptor tyrosine kinase inhibitors (EGFR-TKIs) for non-small cell lung cancer (NSCLC): A protocol of systematic review and network meta-analysis

**DOI:** 10.1097/MD.0000000000023875

**Published:** 2021-01-08

**Authors:** Canfeng He, Ruiting Lin, Jing Zhang, Lingling Sun, Jietao Lin, Lizhu Lin

**Affiliations:** a The First Clinical Medical College, Guangzhou University of Chinese Medicine; b Department of Oncology Center, First Affiliated Hospital of Guangzhou University of Chinese Medicine, Guangzhou, China.

**Keywords:** acneiform, epidermal growth factor receptor tyrosine kinase inhibitors, network meta-analysis, randomized controlled trials

## Abstract

**Background:**

Acneiform eruptions from epidermal growth factor receptor tyrosine kinase inhibitors is a frequent adverse event in non-small cell lung cancer patients but the efficacy of its treatment including antibiotics, corticosteroid, sunscreen is still poorly understood.

**Methods:**

Eight electronic databases (PubMed, EMBASE, ClinicalTrials.gov, etc) will be searched from inception to April 2020. Risk of bias of randomized controlled trials will be assessed in terms of the Risk of Bias 2 (RoB 2) tool. Eligible randomized controlled trials will be enrolled for a Bayesian network meta-analysis using R software.

**Results:**

This study is still ongoing and the results will be submitted and published in a peer-reviewed scientific journal.

**Conclusion:**

We hope the results of this study will provide reliable evidence for the management of acneiform due to epidermal growth factor receptor tyrosine kinase inhibitors for non-small cell lung cancer.

**Ethics and dissemination:**

Ethical approval is not applicable for this study is based on published trials.

**Protocol registration number:**

CRD42020206724

## 1 Introduction

Epidermal growth factor receptor tyrosine kinase inhibitors (EGFR-TKIs) have been widely used in the treatment of non-small cell lung cancer (NSCLC), while an acneiform rash is one of the most common adverse events in patients taking them.^[1]^ Such eruptions are often manifested as pustular lesions in scalp, face, upper chest, and back with or without pruritus or tenderness, mostly appearing in 1 to 2 weeks after using EGFR-TKIs.^[2,3]^ Unfatal as acneiform is 9, clinical benefits of EGFR-TKIs are undermined for its damage to patient's appearance, quality of life, and treatment adherence.^[4]^

Management of acneiform rash caused by EGFR-TKIs from general guidelines and recommendations consist of prophylactic and reactive strategies. Antibiotics are the most studied and recommended agents during prophylactic treatment, and a previous meta-analysis suggests that pre-emptive oral use of tetracyclines before the onset of anti-EGFR treatment could lower acneiform incidence by 50%.^[5]^ Several studies also evaluate the prophylactic efficacy of adapalene, skin moisturizer, or sunscreen but observed different results.^[6,7]^ Reactive regimens mainly compose of topical and systemic corticosteroid or antibiotics in accordance with acneiform severity grading by common terminology criteria for adverse events (CTCAE) developed by National Cancer Institute. However, these management principles mostly gleaned from consensus, expert opinions, or small sample trials, and few recommendations are evidence based.^[8]^ Therefore, we propose to conduct a systematic review and network meta-analysis of randomized controlled trials (RCTs) to compare the efficacy of treatment for acneiform eruptions due to EGFR-TKIs.

## 2 Materials

### 2.1 Study registration

The study protocol developed based on preferred reporting items for systematic review and meta-analysis protocols (PRISMA-P) has been registered on PROSPERO (International Prospective Register of Systematic Reviews) (CRD42020206724). If an amendment of the protocol is required, it will be documented in PROSPERO.

### 2.2 Eligibility criteria

#### 2.2.1 Type of research

Randomized controlled trials with or without blind methods will be included unless relevant data cannot be accessible and extracted. Besides, any reviews, guidelines, letters, systematic reviews, meta-analysis, case reports, comments as well as non-English publications will be excluded.

#### 2.2.2 Participants

Patients who underwent prior EGFR-TKIs (gefitinib, erlotinib, afatinib, etc) with NSCLC diagnosis developed secondary acneiform.

#### 2.2.3 Interventions

Pharmacological interventions (like corticosteroid or antibiotics), cosmetics (like skin moisturizer or sunscreen), and other potential therapeutic approaches are demanded in the treatment group while placebos in the control group. No limitations are imposed on dosage, onset time of interventions, or duration.

#### 2.2.4 Outcomes

The primary outcome is rash severity, defined as grade 2 to grade 4 rash according to National Cancer Institute Common Terminology Criteria for Adverse Events, NCT-CTCAE), and the additional outcome is the dermatology-related quality of life.

### 2.3 Literature search strategy

Literature searches will be conducted in PubMed, Embase, Web of Science (WOS), Cochrane Central Register of Controlled Trials (CENTRAL), and scopus from inception to April 2020 with language restriction to English. Additionally, clinical trial information from Clinical Trials.gov and meeting abstracts from the American Society of Clinical Oncology (ASCO) and European Society for Medical Oncology (ESMO) will also be searched for ongoing or complete but unpublished studies. The search strategy used in PubMed database is shown in Table 1. Similar strategies will be adapted and adopt for searching other database.

**Table 1:** The search strategy for PubMed.

No.	Search item
1#	“Acneiform eruptions”[mesh terms] or “exanthema”[mesh terms] or “folliculitis”[mesh terms] or “drug eruptions”[mesh terms]
2#	“Acne∗”[title/abstract] or “acneiform”[title/abstract] or “eruption∗”[title/abstract] or “rash∗”[title/abstract] or “exanthem∗”[title/abstract] or “comedone∗”[title/abstract] or “pustul∗”[title/abstract] or “papul∗”[title/abstract] or “macul∗”[title/abstract] or “folliculit∗”[title/abstract]
3#	#1 or #2
4#	“Erbb receptors/antagonists and inhibitors”[mesh terms] or “gefitinib”[mesh terms] or “erlotinib hydrochloride”[mesh terms] or “icotinib”[mesh terms] or “afatinib”[mesh terms] or “dacomitinib”[mesh terms] or “osimertinib”[mesh terms]
5#	“Epidermal growth factor receptor”[title/abstract] or “EGFR”[title/abstract] or “HER1”[title/abstract] or “HER-1”[title/abstract] or “erbb-1”[title/abstract] or “erbb1”[title/abstract]
6#	“Tyrosine kinase inhibitor∗”[title/abstract] or “TKI”[title/abstract] or “tkis”[title/abstract] or “antagonist∗”[title/abstract] or “inhibitor∗”[title/abstract]
7#	#5 and #6
8#	“Gefitinib”[title/abstract] or “iressa”[title/abstract] or “ZD1839”[title/abstract] or “ZD 1839”[title/abstract] or “ZD-1839”[title/abstract] or “erlotinib”[title/abstract] or “osi 774”[title/abstract] or “OSI774”[title/abstract] or “OSI-774”[title/abstract] or “tarceva”[title/abstract] or “icotinib”[title/abstract] or “afatinib”[title/abstract] or “bibw 2992”[title/abstract] or “gilotrif”[title/abstract] or “BIBW2992”[title/abstract] or “dacomitinib”[title/abstract] or “PF-00299804”[title/abstract] or “PF299804”[title/abstract] or “PF-05199265”[title/abstract] or “PF05199265”[title/abstract] or “osimertinib”[title/abstract] or “tagrisso”[title/abstract] or “AZD9291”[title/abstract] or “AZD 9291”[title/abstract] or “AZD-9291”[title/abstract]
9#	#4 or #7 or #8
10#	“Carcinoma, non-small-cell lung”[mesh terms]
11#	“lung”[title/abstract] or “pulmon∗”[title/abstract] or “pneumo∗”[title/abstract] or “bronchus”[title/abstract] or “bronchogenic”[title/abstract] or “bronchial”[title/abstract] or “bronchiolo alveolar”[title/abstract] or “bronchiolo-alveolar”[title/abstract] or “bronchioloalveolar”[title/abstract] or “bronchiolar”[title/abstract] or “alveolar”[title/abstract]
12#	“Cancer∗”[title/abstract] or “carcinoma∗”[title/abstract] or “neoplasm∗”[title/abstract] or “malignan∗”[title/abstract] or “tumo?r∗”[title/abstract] or “adenocarcinoma∗”[title/abstract]
13#	#11 and #12
14#	“Non-small cell∗”[title/abstract] or “nonsmall cell∗”[title/abstract] or “non small cell∗”[title/abstract]
15#	#13 and #14
16#	“Non-small-cell lung cancer”[title/abstract] or “non-small-cell lung carcinoma”[title/abstract] or “non-small cell lung cancer”[title/abstract] or “non-small cell lung carcinoma”[title/abstract] or “non small-cell lung Cancer”[title/abstract] or “non small-cell lung carcinoma”[title/abstract] or “non small cell lung cancer”[title/abstract] or “non small cell lung carcinoma”[title/abstract] or “NSCLC”[title/abstract]
17#	#10 or #15 or #16
18#	(Randomized controlled trial [pt] or controlled clinical trial [pt] or randomized [tiab] or placebo [tiab] or clinical trials as topic [mesh: no exp] or randomly [tiab] or trial[ti]) not (animals [mh] not humans [mh])
19#	English[language]
20#	#3 and #9 and #17 and #18 and #19

### 2.4 Study selection

Titles and abstracts will be scanned independently by 2 authors so that potential eligible studies will be identified when they met the following criteria. Any disagreement will be resolved through a discussion with a third author.

### 2.5 Data extraction and management

A standard form involving study information (author, publication year, registration ID and study design and sample size), patient characteristics (age, sex, cancer diagnosis, and EGFR-TKIs usage), interventions’ information (name, dosage, and duration) and outcomes will be filled out by 2 authors independently. Discrepancies will be resolved by a discussion with a third author.

### 2.6 Assessment of risk of bias

Included RCTs will be evaluated by 2 authors independently for risk of bias using “risk of bias 2 (RoB 2)”, recommended by the Cochrane Collaboration.^[9]^ The tool composes of 5 domains, including randomization process, deviations from the intended interventions, missing outcome data, measurement of the outcome, and selection of the reported result. And risk of bias judgement will be graded as low risk, some concerns, or high risk.

### 2.7 Statistical analysis

R software (version 3.5.3) will be used to carry out the Bayesian network meta-analysis, and *P*-value <.05 is considered statistically significant. Outcomes will be reported as risk ratios (RRs) with their 95% confidence intervals. A network plot will be drawn to present the geometry of interventions across studies. I2 statistic is calculated to assess the heterogeneity and if its value is greater than 50%, which indicates significant heterogeneity. Sensitivity analysis will be used to detect possible explanations for the source of heterogeneity. The probability of the ranking of interventions will be evaluated based on the surface under the cumulative ranking curve (SUCRA) with a larger SUCRA value indicating better efficacy. If quantitative analysis is inappropriate due to data limitation or high heterogeneity from unknown sources, our meta-analysis will be replaced by a narrative summary instead.

### 2.8 Subgroup and sensitivity analysis

We will conduct subgroup analysis to explore the difference between type of EGFR-TKIs when sufficient studies are available. Sensitivity analysis will be also performed for assessing robustness of pooled results and identifying the potential source of heterogeneity via excluding each literature separately.

### 2.9 Publication bias

Funnel plot will be presented to explore potential publication bias with Egger test for testing the asymmetry of the funnel plot when 10 or more studies are included.

### 2.10 Assessment of evidence quality

Quality of evidence for outcomes will be appraised using the grading of recommendations assessment, development and evaluation (GRADE) tool where levels of evidence are defined as very low, low, medium, or high.

## 3 Discussion

Most patients with advanced NSCLC receiving EGFR-TKIs experience drug-related toxicities where acneiform is the common complaint.^[10]^ Multiple versions of guidelines of management^[1,11–14]^ of this kind of rash are accessible, whereas most are developed based on evidence from single-arm trials, expert consensus, case reports or retrospective cohort studies.^[15]^ The efficacy of those interventions recommended to prevent or reduce acneiform still remains unclear. Our systematic review and network meta-analysis are designed to will investigate the role and ranking of these interventions based on evidence of RCTs, which may provide practical guidance for managing acneiform eruptions caused by EGFR-TKIs in NSCLC.

## Author contributions

**Conceptualization:** Lingling Sun, Jietao Lin.

**Investigation:** Canfeng He, Jietao Lin.

**Methodology:** Canfeng He, Jing Zhang.

**Project administration:** Canfeng He, Ruiting Lin.

**Supervision:** Jietao Lin, Lizhu Lin.

**Writing – original draft:** Canfeng He.

**Writing – review & editing:** Canfeng He, Ruiting Lin.
